# Evaluation of Cytotoxic Potentials of Novel Synthesized Chalconeferrocenyl Derivative against Melanoma and Normal Fibroblast and Its Anticancer Effect through Mitochondrial Pathway 

**DOI:** 10.22037/ijpr.2020.113949.14578

**Published:** 2021

**Authors:** Ahmad Salimi, Mozhgan Ghasempour, Shabnam Farzaneh, Farzad Khodaparast, Parvaneh Naserzadeh, Afshin Zarghi, Jalal Pourahmad

**Affiliations:** a *Department of Pharmacology and Toxicology, School of Pharmacy, Ardabil University of Medical Sciences, Ardabil, Iran. *; b *Department of Toxicology and Pharmacology, School of Pharmacy, Shahid Beheshti University of Medical Sciences, Tehran, Iran. *; c *Department of Medicinal Chemistry and Nuclear Medicine, School of Pharmacy, Shahid Beheshti University of Medical Sciences, Tehran, Iran.*

**Keywords:** Melanoma, Cyclooxygenase-2 Inhibitor, Mitochondria, Apoptosis, Cancer

## Abstract

The treatment of melanoma is still challenging and therefore identification of novel agents is needed for its better management. Our previous study suggested that cyclooxygenase-2 (COX-2) would be a novel target for treatment of several cancers. In the present study, we searched selective cytotoxicity and mitochondria mediated apoptosis of novel synthesized chalconeferrocenyl derivative (1-Ferrocenyl-3-(dimethylamino)-3-(4-methylsulfonylphenyl) propan-1-one) (FDMPO) as a COX-2 inhibitor on normal and melanoma cells and their mitochondria. For this purpose, we evaluated the cellar parameters such as cytotoxicity, apoptosis% *versus* necrosis%, activation of caspase-3 and ATP content, and also mitochondrial parameters such as reactive oxygen species formation, mitochondrial swelling, mitochondrial membrane potential decline, mitochondrial membrane integrity, and cytochrome C release. Our results showed FDMPO could selectively induce cellular and mitochondrial toxicity (up to 50 µM) on melanoma cells and mitochondria without any toxic effects on normal fibroblast and their mitochondria. Taken together, the results of this study suggest that mitochondria are a potential target for the melanoma. Selective inhibition of mitochondrial COX-2 could be an attractive therapeutic option for the effective clinical management of therapy-resistant melanoma.

## Introduction

Cyclooxygenases (COXs) are expressed in different cell types. COXs are essential enzymes for transformingarachidonic acid to prostaglandin (PG) G2 and afterward to PGH_2_, which is a parent compound for the building of prostanoids, including prostacyclins, thromboxanes and PGs (1). There three main of COX isozymes such as COX-1, COX-2 and COX-3 (1). The COX-2 enzymes are induced and increased during inflammation process. The expansion of COX-2 enzymes has been reported in several types of cancer, such as melanoma, leukemia, colon, breast, prostate and cancer, which is closely linked with chronic inflammation (2-8). Remarkably, the chronic administration of COXs inhibitors have been indicated to decrease the progression and incidence of several cancer (9). Previous studies have been reported that COX-2 isozymes are overexpressed in several tumoral cells and is related with development of cancer, as well as resistance of cancer cells to conventional therapies such as chemotherapy and radiotherapy (10). Recently, celecoxib as a selective COX-2 inhibitors showed a promising effects as an anticancer drug and chemopreventive agents against different cancers (11). According to previous findings, COX-2 inhibitors agents may be a potential therapeutic target for the therapy of cancer.

Notwithstanding advances in surveillance and treatment in melanoma. Annually, this cancer continues to claim approximately 9,000 lives in the US (12). The occurrence of melanoma is growing worldwide. Despite advances in the treatment and surveillance of melanoma, the prognosis of this disease in the patients with advanced metastatic melanoma or high-risk remains poor (13). Surgery followed by adjuvant therapy such as cancer vaccines and interferon-α is the standard treatment for patients with primary melanoma with or without regional metastases to lymph nodes. When the patients progress to the final stage or stage IV metastatic melanoma, the survival is lower than one year. Standard treatment with chemotherapy yields low response rates (13). New strategies for the treating melanoma are cytokine therapy with IL-2, but chemotherapy with cytokine is accompanied by intensive toxicities that require the patient to be hospitalized for support during treatment (14). Epidemiological studies showed that prolonged COX-2 inhibition might offer some protection against some other malignancies (8). A potential role of COX-2 in melanoma progression is also not unlikely since COX-2 is frequently expressed in malignant melanomas, and its inhibition may prevent melanoma progression (15). 

The importance of mitochondria has recently become a focal point of cancer research (16). Oxidative phosphorylation (OXPHOS) in the inner membrane of mitochondria has a prominent role in advanced melanoma (17). It has been reported that targeting mitochondria may have efficacy for the treatment of melanoma (17). Previous studies showed COX-2 localization in several cancer cell lines with a similar distribution pattern by confocal microscopy in mitochondria (18). Also, immunoblot analysis of COX-2 in cytosolic and mitochondrial fractions confirmed the localization of COX-2 to mitochondria in tumoral cells (18). Botti et al, reported that COX-2 expression relates with and modulates programmed death receptor 1 (PD-1) expression in melanoma cells (8). Therefore, targeting mitochondria and COX-2 inhibition may be a potential treatment to prevent melanoma progression. 

## Experimental

*Chemicals*

MTT (3-[4,5-dimethylthiazol-2-yl]-2,5-diphenyltetrazolium bromide), rotenone (Rot), 4-2-hydroxyethyl-1-piperazineethanesulfonic acid (HEPES), dimethyl sulfoxide (DMSO), cyclosporin A (Cs.A), D-mannitol, dithiobis-2-nitrobenzoic acid (DTNB), thiobarbituric acid (TBA), 2′,7′-dichlorofluorescein diacetate (DCFH-DA), tetramethoxypropane (TEP), reduced glutathione (GSH), sodium succinate, malondialdehyde (MDA), Tris–HCl, n-butanol, sulfuric acid, pyruvate, malate, sucrose, ethylene glycol-bis (2-aminoethylether)- N,N,N′,N′-tetraacetic acid (EGTA), KCl, Na_2_HPO_4_, collagenase, MgCl_2_, potassium phosphate, butylated hydroxytoluene (BHT), ethylene ediamine tetra acetic acid (EDTA), rhodamine 123 (Rh 123), Bovine serum albumin (BSA), and coomassie blue were purchased from Sigma Chemical Co. (St. Louis, MO, USA). 

*Chemistry*

The synthesis of target compound 1-Ferrocenyl-3-(dimethylamino)-3-(4-methylsulfonylphenyl) propan-1-one was accomplished by our previously published method (19). In our previous study, this compound having ferrocene motif and methyl sulfonyl COX-2 pharmacophore showed high potency for COX-2 inhibitory and cytotoxicity effects. 

*Experimental data*

*1-Ferrocenyl-3-(dimethylamino)-3-(4-methylsulfonylphenyl) propan-1-one*

The mixture of α, β-unsaturated carbonyl compound (2 mmol) and dimethylamine (2 mmol) was added to a flask immersed in the water bath of an ultrasonic cleaner room temperature controlled by circulated water for 6 hours. After completing the reaction, the crude product was washed with petroleum ether to remove the excess of amines, and a few unreacted α, β -unsaturated carbonyl compounds, and the final product was purified by crystallization (10% EtOAc in light petroleum ether). 

Red solid; decomp: 153 °C; IR (KBr): 1652 (C=O), 1311, 1152 (SO_2_); LC-MS (ESI) *m/z*: 440 (M^+ ^+ 1); H^1^NMR (CDCl_3_): 2.23 (s, 6H, N-C*H*_3_), 2.99 (s, 3H, SO_2_C*H*_3_), 3.15 (m, 1H, C*H*_2_), 3.33 (m, 1H, C*H*_2_), 4.02 (s, 5H, Fc), 4.15 (bs, 1H, C*H*), 4.48 (s, 2H, Fc), 4.72 (s, 2H, Fc), 7.58 (d, 2H, 4-methylsulfonylphenyl *H*_3_ & *H*_5_,* J *= 7.9 Hz), 7.91 (d, 2H, 4-methylsulfonyl phenyl *H*_2_ & *H*_6_,* J *= 7.9 Hz); C^13 ^(CDCl_3_): 42.9, 43.4, 44.5, 65.1 (aliphatic), 69.2, 69.2 (ortho – C_5_H_4_), 69.7 (C_5_H_5_), 72.4, 72.4 (meta – C_5_H_4_), 78.8 (ipso – C_5_H_4_), 127.3, 129.4, 139.4, 147.3 (aromatic), 201.4 (C=O); Anal. Calculated for C_22_H_25_FeNO_3_S: C, 60.14; H, 5.74; N, 3.19. Found: C, 60.34; H, 5.96; N, 3.01.

*Melanoma Tumor Preparation*

Melanoma was inoculated intra dermally in NMRI adult mice with F10 melanoma cells. Briefly, the mice were anesthetized by a combination of xylazine and ketamine administered via intraperitoneal (i.p.) injections and then using a scalpel. The cells were placed underneath the skin, and after all these parts were sutured. The tumor size was measured every 3 days with a digital caliper. Their volume calculated based on O’reilly *et al. *(1997) as follows: 52 v= (tumor weights) 2 (tumor length) 0.52. In the posterolateral part of the body, a small incision was done, and a part of the tumor was extracted and divided into small parts (about 2 mm each). The melanoma and tissues were immediately put into ice-cold RPMI-1640 supplemented with 100 U/mL penicillin G and 100 µg/mL streptomycin. Then, the samples were rinsed with sterile phosphate-buffered saline (PBS) twice and cut into small fragments. Then, the fragments were incubated with a collagenase of 1% in a gently shaking water bath for one h at 37 °C. After passed through a 38 µm mesh sieve, the resulting cell suspension was washed twice and centrifugated at a speed of 300 g × 10 min. Then the pellet was diluted to 1 × 10^6^ cells/mL and incubated in RPMI 1640 containing supplemented with 10% FBS, in 37 °C with 5% CO_2_ (20). 

*Cell treatments*

Melanoma cells and normal fibroblast (10^6^ cells) were cultured in RPMI 1640 medium supplemented with 10% FBS at 37 °C with 5% CO2 in a humidified atmosphere for 12 h. FDMPO was freshly prepared before use and dissolved in DMSO 0.05%. Melanoma cells and normal fibroblast were incubated with or without the treatment of FDMPO (0, 5, 10, 25, 50 and 100 µM) and DMSO 0.05% as control, and all toxicity parameters were evaluated after 12 h. 

*Cytotoxicity Assay*

Melanoma cells and normal fibroblast (10^4 ^cells/well) were exposed to various concentrations of FDMPO (0, 5, 10, 25, 50, and 100 µM). Cell viability at 12 h was determined by MTT with final concentration of 0.5 mg/mL. After 4 h of exposure by adding 100 µL DMSO, the purple-blue MTT formazan precipitate was dissolved, and the absorbance was measured at 570 nm using an enzyme-linked immunosorbent assay (ELISA) reader (Tecan, Rainbow hermo, Austria) (21). 

*Caspase 3 Activity*

Caspase-3 colorimetric assay kit (R&D Systems Inc., Minneapolis, MN, United States) was used to measure the caspase activity in the cell lysates. Melanoma cells and normal fibroblast were exposed to IC50 12 h of FDMPO (50 µM). After treatment, the cells were lysed in a buffer mixture (50 mM Tris-HCl (pH 7.4), 2 mM DTT, 1 mM EDTA, 10 mM digitonin, and 10 mM EGTA). The activation of caspase 3 was estimated by the hydrolysis of substrate peptide, Ac-DEVD-pNA to p-nitroaniline, by caspase 3. p-nitroaniline has a high absorbance at 405 nm. The absorbance was p-nitroaniline measured using an ELISA reader (Tecan, Rainbow hermo, Austria) at 405 nm. Three independent experiments were run for caspase-3 activity determination (21).

*Apoptosis versus Necrosis*

Apoptosis was identified using Annexin V-FITC Apoptosis Kit (K101 BioVision, USA). Briefly, cells were treated with IC50 of FDMPO. After 12 h, the cells were re-suspended in the 500 μL binding buffer. FITC-conjugated annexin V and PI were added, and after 5 min incubation, samples were analyzed on a flow cytometer (Cyflow Space-Partec, Germany).

*Preparation of Mitochondria*

The melanoma and normal tissues were dissected and cut into slices (1 mm) using surgical scissors and cleared from blood vessels, and homogenized with a glass homogenizer in a 10-fold volume of the isolation buffer (225 mM D-mannitol, 75 mM sucrose, and 0.2 mM EDTA, pH 7.4) in an ice-cold bath. The homogenate was centrifuged at 1000 ×g for 10 min, and the pellet was removed. The supernatant containing mitochondria was centrifuged at 10000 ×g for 10 min at 4 °C. The protein content in mitochondria was determined using the Bradford assay was determined (22). Protein concentration in the suspension was 1000 µg/mL. The number of mitochondria for each test was 1000 µg/mL. The isolated mitochondria from both groups were treated with various concentrations of FDMPO (0, 5, 10, 25, 50, and 100 µM) for the assessment of succinate dehydrogenases activity, and for the other testes were exposed with ½ IC50, IC50, and 2IC50 at 37 °C for 1 h.

*Succinate Dehydrogenases Activity*

The activity of succinate dehydrogenases or SDH (mitochondrial complex II) was measured by the reduction of MTT. Briefly, 100 µL of mitochondrial suspensions (containing 100 µg protein mitochondria, 70 mM sucrose, 230 mM mannitol, 3 mM HEPES, 2 mM Tris-phosphate, 5 mM succinate, and 1 µM of rotenone with pH 7.4.) was incubated with various concentrations of FDMPO (0, 5, 10, 25, 50 and 100 µM) at 37 °C for 1hr. Then, 25 µL of 0.4% MTT was added to the medium at 37 °C for 30 min. The product of formazan crystals was dissolved in 100 µL DMSO, and the absorbance at 570 nm was measured with an ELISA reader (Tecan, Rainbow hermo, Austria (23).

*Mitochondrial Swelling Assay*

Mitochondrial swelling was monitored as the changes in absorbance at 540 nm. Incubations with various concentrations of FDMPO (25, 50, and 100 µM) were carried out at 37 °C with 100 µg of mitochondrial protein/mL in the swelling buffer containing 70 mM sucrose, 230 mM mannitol, 3 mM HEPES, 2 mM Tris-phosphate, 5 mM succinate and 1 µM of rotenone with pH 7.4. Mitochondrial swelling was measured spectrophotometrically in 60 min (15, 30, 45, and 60 min). Mitochondrial swelling results in a decrease in absorbance monitored at 540 nm (23). 

*Mitochondrial ROS Formation Assay*

Briefly, purified mitochondria (1000 µg protein/mL) were isolated and were placed in respiration buffer (0.32 mM sucrose, 10 mM Tris, 20 mM Mops, 50 μM EGTA, 0.5 mM MgCl_2_, 0.1 mM KH_2_PO_4_ and 5 mM sodium succinate 10 µM DCFH-DA with pH 7.4) with various concentrations of FDMPO (25, 50 and 100 µM). Fluorescence intensity of DCFH-DA was measured using fluorescence spectrofluorometer at an excitation/emission wavelength 488 nm and 527 nm in duration 60 min (15, 30, 45, and 60 min) (23). 

*Mitochondrial MMP Collapse Assay*

Briefly, the mitochondrial fractions (1000 µg protein/mL) were incubated with various concentrations of FDMPO (25, 50, and 100 µM) in MMP assay buffer (220 mM sucrose, 68 mM D-mannitol, 10 mM KCl, 5mM KH_2_PO_4_, 2 mM MgCl_2_, 50 μM EGTA, 5 mM sodium succinate, 10 mM HEPES, 2 μM Rotenone, 10 µM rhodamine 123), at 37 ºC for an hour. The fluorescence was monitored using spectrofluorometer at the excitation and emission wavelength of 490 nm and 535 nm, respectively, in 60 min (15, 30, 45 and 60 min) (23). 

*Cytochrome c Release Assay*

Isolated mitochondria were incubated in 1.5-mL Eppendorf tubes within buffer assay (140 mM KCl, 10 mM NaCl, 2 mM MgCl_2_, 0.5 mM KH_2_PO_4_, 20 mM HEPES, 0.5 mM EGTA; pH 7.2). Inhibitor of MPT pore, cyclosporine A at the final concentration 5 µM and anti-oxidant BHT at the final concentration 5 µM were added 15 min before the addition of FDMPO (IC50). After an hour, the tubes were centrifuged at 10,000 g × 10 min. The supernatant contained the cytochrome c released from the mitochondria (cytosolic fraction), and the pellet consisted of the mitochondrial fraction. The concentration of cytochrome c was determined by using the Quantikine Rat/Moue Cytochrome c Immunoassay kit (Minneapolis, Minn) according to the manufacturer’s instructions (24). 

*Statistical Analysis*

The results were analyzed using Graph Pad Prism (version 5, Graph Pad Software Inc., La Jolla, CA, USA). Results are presented as mean ± SD. Assays were performed in triplicate, and the mean was used for statistical analysis. Statistical significance was determined using the one-way ANOVA test, followed by the post-hoc Tukey posttest, and two-way ANOVA followed by the posttest Bonferonie. Statistical significance was set at *p* < 0.05. 

## Results

*FDMPO Selectively Induced Cytotoxicity in Melanoma Cancer Cells*

**Figure 1:**
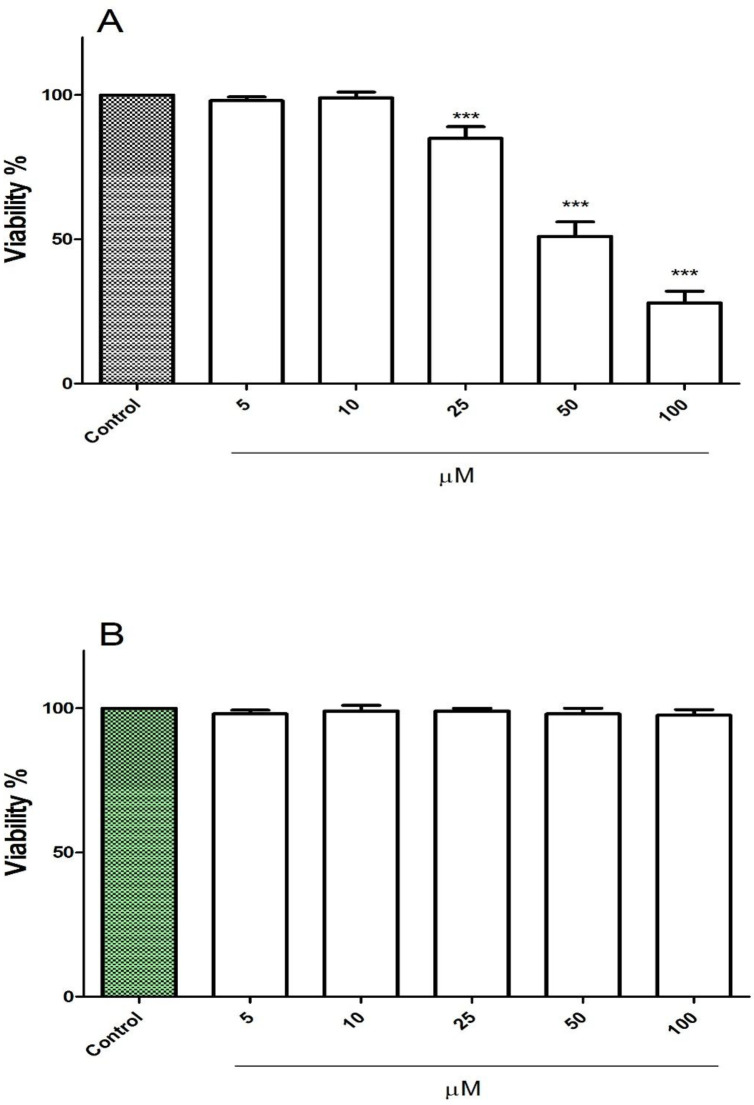
Effect of FDMPO on viability of melanoma cells and normal fibroblcytes. (A) melanoma cells and (B) normal fibroblcytes were treated in with the different concentrations of FDMPO (0, 5, 10, 25, 50 and 100 µM) and cell viability was measured by MTT assay at 12 h. Values were expressed as mean ± SD of three separate determinations. (***p < 0.001 *vs.* untreated control with FDMPO). Assays were performed in triplicate (n = 3).

The cytotoxic effect of FDMPO at various concentrations 0, 5, 10, 25, 50, and 100 µM was tested against melanoma cells and normal fibroblast. Our results showed that 1 FDMPO was more cytotoxic on melanoma cells compared to normal fibroblast. As shown in Figure 1A, FDMPO at concentrations of 50 and 100 µM significantly (*p* < 0.001) reduced cell viability, while no cytotoxicity was showed at these concentrations on normal fibroblast (Figure 1B). 

*FDMPO Selectively Induced Apoptosis in Melanoma Cancer Cells*

**Figure 2:**
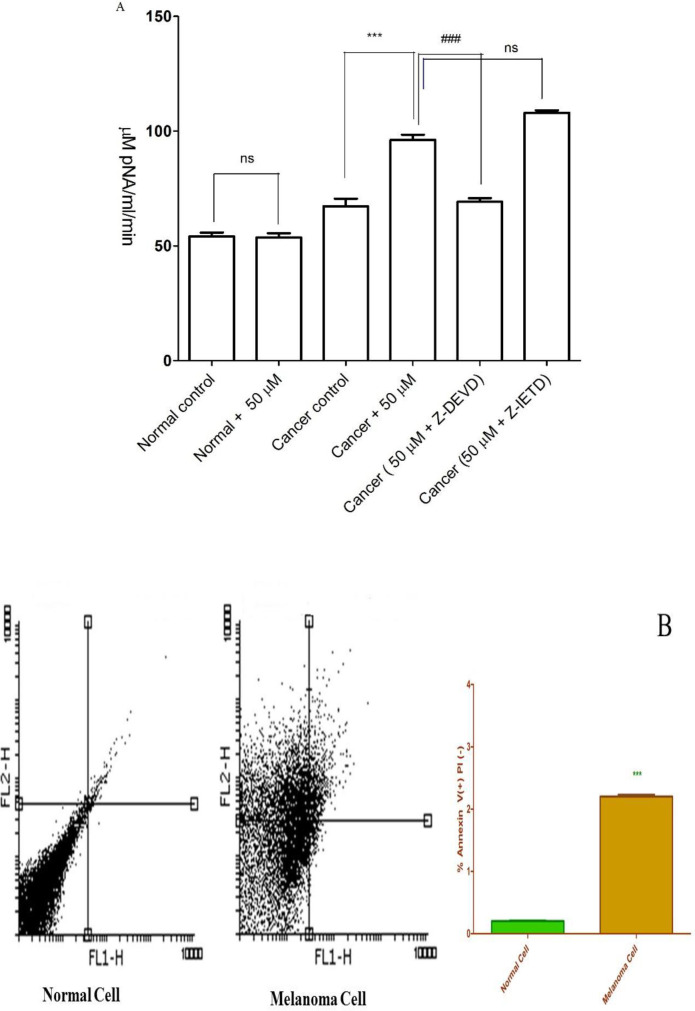
Effect of FDMPO on caspase-3 activation and apoptosis and necrosis% in melanoma cells and normal fibroblcytes.  (A) Cells (10^6^ cells/mL) were treated with 50 µM of FDMPO at 12 h. Caspase-3 activity was determined by Sigma-Aldrich kit. The kit determines produced pNA that is released from the interaction of caspase-3 and AC-DEVD-pNA (peptide substrate). FDMPO significantly increased the activity of caspae-3 in melanoma cells but not in human normal fibroblcytes. Z-IETD a caspase 8 inhibitor not affected on caspase 3 activation. Values are expressed as mean ± SD of three separate experiments (n = 5). ^***^Significant difference in comparison with cancerous control (p<0.001) and ^###^Significant difference in comparison with 50 µM new chalconeferrocenyl derivative (p<0.001). (B) Effects of FDMPO on apoptosis in melanoma cells and normal fibroblcytes. FDMPO-induced apoptosis in melanoma cell but not in normal fibroblcytes at IC50 concentrations (50 μM) within 12 h. Assays were performed in triplicate (n = 3).

Caspase-3 is activated in the cell under apoptosis signaling through extrinsic (death ligand) and intrinsic (mitochondrial) pathways. As shown in Figure 2A, FDMPO (50 µM) significantly increased the activity of caspase-3 as a final apoptosis mediator in melanoma cells. Increased caspase-3 activity at 50 µM of FDMPO was not observed in normal fibroblasts. To understand the upstream mechanism involved in FDMPO-induced caspase-3 activation we examined the pretreating effect of Z-IETD a caspase 8 inhibitor and Z-DEVD a caspase 3 inhibitor. Our results showed that Z-IETD as a caspase 8 inhibitor has no effect FDMPO-induced caspase-3 activation, suggesting that FDMPO activates a mitochondria-mediated intrinsic pathway apoptosis in melanoma cells but no normal fibroblasts. 

*FDMPO Selectively activated Caspase 3 in Melanoma Cancer Cells*

The activity of caspae-3, as a critical apoptosis mediator, significantly (*p* < 0.01) enhanced in melanoma cells after treatment with FDMPO at 12 h; however, this effect was not observed on normal fibroblasts (Figure 2B). 

*FDMPO Selectively Decrease SDH Activity in Cancerous Mitochondria*

**Figure 3:**
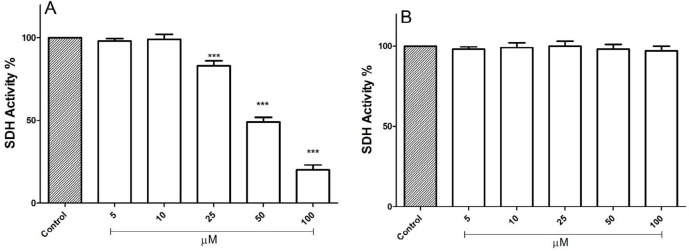
Effect of FDMPO on succinate dehydrogenase activity in melanoma and normal fibroblcytes mitochondria. This figure demonstrates the effect of the FDMPO on succinate dehydrogenase activity in both melanoma (A) and normal fibroblcytes mitochondria (B). Mitochondrial succinate dehydrogenase activity was measured by MTT assay within 1 h after FDMPO exposure. Values were expressed as mean ± SD of three separate determinations. ^(***^p < 0.001 *vs.* untreated control with FDMPO. Assays were performed in triplicate (n = 3)

Evaluations of FDMPO for potential activity on mitochondria obtained from both melanoma cells and normal fibroblasts were carried out by studying the inhibitory effects of this compound on succinate dehydrogenase activity using the MTT assay. FDMPO (25, 50, and 100 µM) strongly inhibited succinate dehydrogenase activity only in melanoma mitochondria but not in normal fibroblast mitochondria (Figures 3A-3B). 

*FDMPO Selectively Increased ROS Formation in Cancerous Mitochondria*

**Figure 4:**
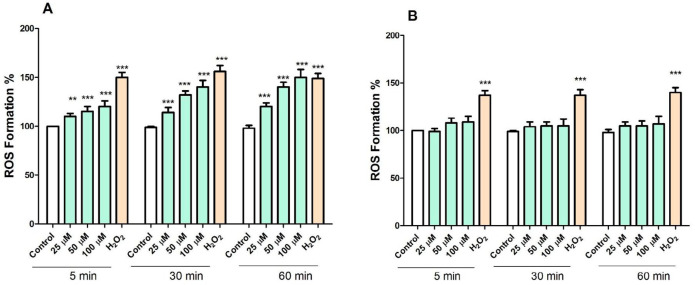
Effect of FDMPO on mitochondrial swelling in melanoma and normal fibroblcytes mitochondria. FDMPO at different concentrations (25, 50 and 100 µM) induced mitochondrial swelling in melanoma (A) but not normal fibroblcytes mitochondria (B). Mitochondrial swelling was monitored by following 540 nm absorbance decrease. Values were expressed as mean ± SD of three separate determinations. ^(**^^*^p < 0.001 *vs.* untreated control with FDMPO. Assays were performed in triplicate (n = 3).

ROS plays a crucial role in cell survival and death. We examined whether the level of ROS in cancerous and normal mitochondria is affected by FDMPO. As shown in Figure 4A, treatment with FDMPO at 25, 50, and 100 µM at 1 h, significantly induced ROS formation (*p* < 0.05) in cancerous mitochondria. These results suggested that FDMPO induced ROS formation might underlie its effect on promoting melanoma cells apoptosis. However, as shown in Figure 4B, treatment with FDMPO at 025, 50, and 100 µM at 1 h did not induce ROS formation in normal fibroblast mitochondria.

*FDMPO Selectively Induced MMP Collapse in Cancerous Mitochondria*

**Figure 5.**
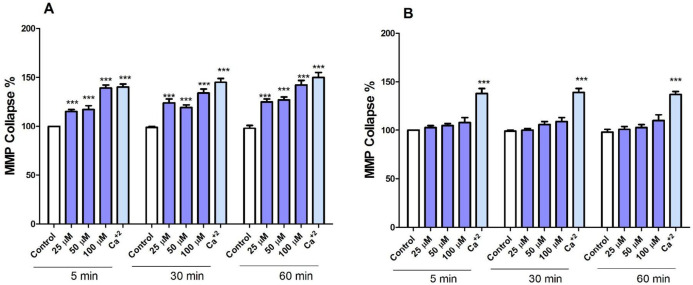
Effect of FDMPO on ROS formation in melanoma and normal fibroblcytes mitochondria. Freshly isolated mitochondria were incubated from both groups with the different concentrations of FDMPO (25, 50 and 100 µM) for 1 h. ROS was measured by DCFH-DA staining with spectrofluorescence method. (A) The ROS formation percentage was significantly increased (*p *< 0.001) by FDMPO in comparison to untreated cancer control. (B) In normal fibroblcytes mitochondria the changes of the ROS formation percentage were not significant. Values were expressed as mean ± SD of three separate determinations. (^***^p < 0.001 *vs.* untreated control with FDMPO. Assays were performed in triplicate (n = 3).

To search for the identification of mechanisms involved in apoptosis, we examined the effects of FDMPO on membrane permeability of mitochondria (ΔΨm) in isolated mitochondria from both groups. Treatment with various concentrations of FDMPO (25, 50, and 100 µM for 1 h) induced a significant decrease in ΔΨm only in melanoma mitochondria (Figure 5A). Treatment with FDMPO (25, 50, and 100 µM for 1 h) did not induce ΔΨm collapse in normal fibroblast mitochondria (Figure 5B). 

*FDMPO Selectively Induced Mitochondrial Swelling in Cancerous Mitochondria*

**Figure 6.**
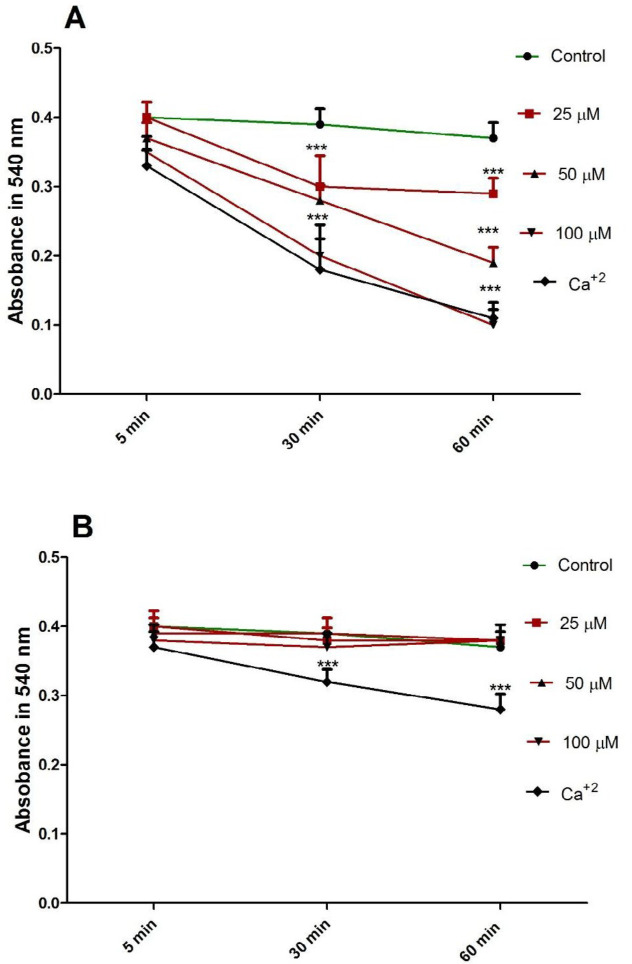
Effect of FDMPO on ΔΨm in melanoma and normal fibroblcytes mitochondria. (A) Freshly isolated mitochondria from both group cells were treated with the different concentrations of FDMPO (25, 50 and 100 µM) for 1 h. ΔΨm was measured spectrophotometrically by rhodamine 123 staining. The presented data revealed that the FDMPO induced a decrease in ΔΨm only in melanoma mitochondria but not in normal fibroblcytes mitochondria (B). Values were expressed as mean ± SD of three separate determinations. (^***^p < 0.00 1 *vs.* untreated control with FDMPO. Assays were performed in triplicate (n =3).

Induction of mitochondrial swelling in isolated mitochondria was monitored by following 540 nm absorbance (A540) decrease. FDMPO (25, 50, and 100 µM for 1h) resulted in an extensive mitochondrial swelling in melanoma mitochondria (Figure 6A). FDMPO addition to normal mitochondria (25, 50, and 100 µM for 1h) not resulted in mitochondrial swelling in normal fibroblast mitochondria (Figure 6B). 

*FDMPO Selectively Induced Cytochrome C Release in Cancerous Mitochondria*

**Figure 7:**
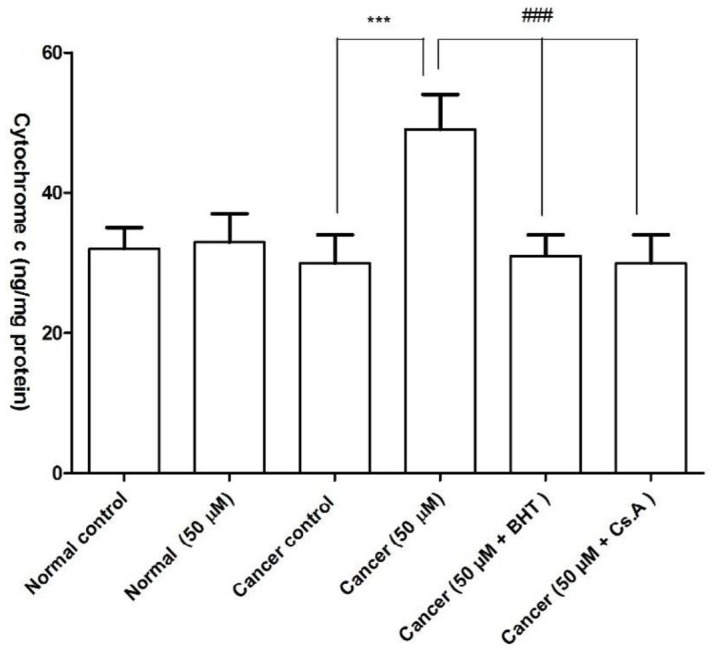
Effect of FDMPO on the cytochrome c release in melanoma and normal fibroblcytes mitochondria. As shown in this figure, pretreatment with FDMPO in cancerous mitochondria significantly induced cytochrome c release BUT NOT in normal fibroblcytes mitochondria. The amount of expelled cytochrome c from mitochondrial fraction into the suspension buffer was determined using human Cytochrome c ELISA kit. Values were expressed as mean ± SD of three separate determinations. (^***^p < 0.001 *vs.* untreated control with normal fibroblcytes mitochondria and ^###^Significant difference in comparison with 50 µM FDMPO (p < 0.001). Assays were performed in triplicate (n = 3)

Our results showed that FDMPO significantly caused mitochondrial swelling and collapse of the mitochondrial membrane potential. These events could result in mitochondrial permeability transition and release of cytochrome c from mitochondria. As shown in Figure 7, FDMPO induced significant (*p* < 0.05) release of cytochrome c on the melanoma mitochondria but not in normal fibroblast mitochondria. Significantly, the pretreatment of FDMPO-treated mitochondria with the MPT inhibitor, cyclosporine A (Cs. A), and ROS scavenger, butylated hydroxyl toluene (BHT) prevented cytochrome c release, indicating the role of oxidative stress and MPT pore opening in melanoma mitochondria. 

**Scheme1:**
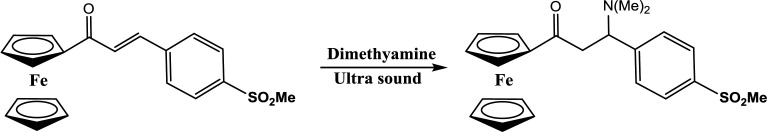
Synthesis of target ferrocene derivative

## Discussion

COX-2 isoenzymes are shown to be over expressed during both inflammation and cancer (9, 25). Over expression of COX-2 in melanoma cancer correlates with the patient’s prognosis and pathological features of primary tumors, and it is demonstrated to be expressed in both metastatic and primary lesions (8, 26-28). Many studies show a role for COX-2 isoenzyme in the modulation and development of different steps of cancer progression (29). Our data demonstrated that COX-2 is involved in the proliferation of melanoma cells since treatment of melanoma cells with the COX-2 inhibitor FDMPO inhibits their proliferation and induces cell death. Also, here, we showed that FDMPO, a novel COX-2 inhibitor, induced selective toxicity in melanoma cells and their isolated mitochondria without any significant effect on normal fibroblasts. This effect is in line with previous works (7, 8).

Defected apoptosis signaling represents a major causative factor in the development and progression of melanoma (30). The ability of melanoma cells to evade the engagement of apoptosis can play a significant role in their resistance to conventional therapeutic regimens (31). We proved that FDMPO selectively induced apoptosis in melanoma cells but not normal fibroblasts. According to accepted models, apoptotic cell death can result from the activation of two different but interrelated molecular cascades. An extrinsic pathway transduces the extracellular stimulus, protein death ligands, through plasmatic membrane (32). Besides, an intrinsic pathway controls and monitors the intracellular environment, the mitochondria, which evaluate the molecular signals leading to death or survival (32). In the intrinsic apoptotic machinery, alterations in key regulators’ expression or components have been associated with different human cancers. For example, in numerous human melanoma samples, reduced expression of Apaf-1 has been observed and correlates with disease progression (33). Soengas et al showed that the frequent transcriptional silencing of Apaf-1 results from the aberrant methylation of the promoter sequences in the gene in metastatic melanomas (34). Our results from caspase-3 activation showed that the intrinsic pathway or mitochondrial pathway is involved in the induction of apoptosis by FDMPO.

Mitochondria play a critical role in regulating the metabolic characteristics of all cells (35). Despite this central role, significant variation in mitochondrial content and function exists in melanoma, often regulated by MITF (melanocyte inducing transcription factor) and PGC-1 (the peroxisome proliferator-activated receptor gamma coactivator 1 alpha). BRAF-driven melanomas with high mitochondrial content exhibit increased oxidative phosphorylation when treated with BRAF inhibitors and are more sensitive to mitochondrial-targeted drugs (36, 37). This subset of melanomas also exhibits increased ROS production and resistance to ROS toxicity (38). A recent study has been shown that mitochondrial dynamic alterations regulate melanoma cell migration and progression (39). Analysis of COX-2 expression in melanoma metastases demonstrates a significant correlation between the percent of expression and progression of melanoma (26). These findings have been suggested that COX-2 expression may become a useful diagnostic tool and a possible therapeutic in melanoma malignancy (26). The above study confirmed that COX-2 is essential for melanoma development in mice (26). Previous studies showed COX-2 localization in several cancer cell lines with a similar distribution pattern by confocal microscopy in mitochondria (18). Our result in isolated mitochondria showed that FDMPO is selective toxic agent on melanoma mitochondria compared to normal fibroblast mitochondria.

 In summary, our results proved that the inhibition of COX-2 could induce selective toxicity in the melanoma cell and suggest that probably mitochondrial COX-2 is a promising therapeutic target for melanoma treatment. By comparing melanoma cells with the normal fibroblast, the results described above allow us to predict that targeting mitochondrial COX-2 could be the key target for the treatment of melanoma. Therefore, our present results may lead to new possibilities in chemoprevention and chemotherapy for melanoma. The limitation of the current study is that these findings must be verified by animal and clinical trials on humans to establish the antitumor effects of FDMPO in melanoma patients.
